# Post COVID-19 symptoms are common, also among young adults in the general population

**DOI:** 10.1038/s41598-023-38315-2

**Published:** 2023-07-12

**Authors:** Ida Mogensen, Sandra Ekström, Jenny Hallberg, Antonios Georgelis, Erik Melén, Anna Bergström, Inger Kull

**Affiliations:** 1 Department of Clinical Science and Education Södersjukhuset, Karolinska Institutet, Stockholm, Sweden; 2 Center for Occupational and Environmental Medicine, Region Stockholm, Stockholm, Sweden; 3 Institute of Environmental Medicine, Karolinska Institutet, Stockholm, Sweden; 4 Sachs’ Children and Youth Hospital, Södersjukhuset, Stockholm, Sweden

**Keywords:** Diseases, Medical research, Risk factors, Signs and symptoms

## Abstract

Post coronavirus disease-19 (post COVID-19) is mainly studied in clinical populations and less is known about post COVID-19 in a young general population. The aim of the study is to investigate the prevalence and symptoms of post COVID-19 and its potential risk factors in young adults. Participants from the Swedish population-based birth cohort BAMSE were included (n = 2022, mean age 26.5 years). Post COVID-19 was assessed through a questionnaire and defined as symptoms after confirmed COVID-19 (registry-based or self-reported positive test) lasting for ≥ 2 months. In total, 681 participants had had confirmed COVID-19. Among them, 112 (16.5%) fulfilled the definition of post COVID-19 (17.8% in females, 14.5% in males, p = 0.26). The most common post COVID-19 symptoms were altered smell and taste (68.8%), dyspnea (33.7%) and fatigue (30.4%). Overall, no major risk factors for post COVID-19 were identified except for being bedbound during COVID-19. However, asthma and rhinitis were associated with the post COVID-19 symptom dyspnea, migraine with altered smell and taste, and lower self-rated health with fatigue. In conclusion, post COVID-19 symptoms are common, also among young adults in the general population. Although not life-threatening, it could have a considerable impact on public health due to the high prevalence and long-term symptoms.

## Introduction

The corona virus disease-19 (COVID-19) was declared a pandemic in March 2020 by the World Health Organization (WHO)^1^. During the course of the pandemic, it has become clear that the infection can lead to severe disease and death, and also long-term sequelae labelled “post COVID-19” or “long covid”^2–5^. The reported prevalence of post COVID-19 has been in a wide range from around 6% to close to 100% among those with a confirmed disease^2,6^. A recent review found the prevalence in non-hospitalized patients to be between 7.5–41%^7^, although the study populations (e.g., age range) and definitions of post COVID-19 differed considerably between the different studies which could affect the prevalence^4^. WHO proposed the following definition of post COVID-19 in December 2021: “Post COVID-19 condition is defined as the illness that occurs in people who have a history of probable or confirmed severe acute respiratory syndrome coronavirus 2 (SARS-CoV-2) infection; usually within three months from the onset of COVID-19, with symptoms and effects that last for at least two months”^8^. After the acute infection has resolved, the remaining or upcoming symptoms are considered emanating from tissue injury caused by the virus itself, by the hyperinflammation caused by disease, or by post-infectious conditions related to the disease but not directly caused by the viral effect (for example being bedbound or invasive ventilation)^3^.

Post COVID-19 is generally reported to be more common among females compared to males^6,9–15^. In addition, asthma has, in several studies, been found to associate to post COVID-19^4,10,16^. Moreover, age (> 50 years), smoking, cardiovascular disease, chronic lung disease, obesity, and psychiatric disease have also been associated with a higher risk for post COVID-19 symptoms^4,7,13,14,17–19^. The condition can be present both after severe and mild disease^2,20^, although the prevalence and symptom pattern may differ depending on severity^21–24^.

However, most studies on post COVID-19 are based on clinical populations. Hence, in younger age groups, with predominantly mild disease, post COVID-19 symptoms and risk factors are less studied. The potential post COVID-19 burden in young adults is of importance both for health care providers and public, due to the often long duration of the symptoms and their debilitating effect at an age when it is common to both start a family and be in the beginning of a working life.

In the present study, we characterize the post COVID-19 symptomatology in young adults from a population-based cohort and investigate risk factors and clinical characteristics associated with post COVID-19 condition.

## Results

### Description of the study population

The study population consisted of 2022 individuals with available information on confirmed COVID-19 (i.e. positive test) and post COVID-19 symptoms (n = 1193 (59.0%) women and n = 829 (41.0%) men). The mean age was 26.5 years (range 24.9–27.9). A description of the study population is presented in Table 1.

**Table 1:** Description of the study population (n = 2022).

	Participant data (n = 2022)^a^
Age (n = 2022), mean (SD)	26.5 (0.79)
Sex (n = 2022)	
Females	1193/2022 (59.0)
Males	829/2022 (41.0)
Living area (n = 2022)	
Stockholm County	1551/2022 (76.7)
Outside of Stockholm County	471/2022 (23.3)
Month of answering the questionnaire (n = 2022)	
October 2021	1300/2022 (64.3)
November 2021	406/2022 (20.1)
December 2021	62/2022 (3.1)
January 2022	231/2022 (11.4)
February 2022	23/2022 (1.1)
Education (n = 1948)	
Elementary school	37/1948 (1.9)
High school/upper secondary school	557/1948 (28.6)
University/College < 3 years or folk high school	304/1948 (15.6)
University ≥ 3 years	1050/1948 (53.9)
Occupation (n = 1953)	
Student	546/1953 (28.0)
Worker	1270/1953 (65.0)
Other	137/1953 (7.0)
Confirmed COVID-19 (n = 2022)^b^	681/2022 (33.7)

^a^Unless otherwise indicated, data are expressed as number/total number (%) The total number is smaller for some variables owing to missing data.

^b^Confirmed COVID-19: self-reported SARS-CoV-2 positive PCR-test, antigen test or IgG test before vaccination and/or positive PCR-test in the database SmiNet (a national register covering all positive PCR-tests for SARS-CoV-2 conducted in Sweden).

The prevalence of confirmed COVID-19 was 33.7% (n = 681), with no difference among females (34.0%) and males (33.3%). Among them, around half (n = 356, 52.3%) were identified through the national SmiNet register^25^, whereas the others were based on self-reported positive polymerase chain reaction (PCR), antigen, or antibody test (antibody test taken before vaccination) (Supplementary Fig. 1 online). In total, 5 (< 1%) of the participants with confirmed COVID-19 reported being admitted to hospital. There were few differences in sociodemographic factors, lifestyle, or chronic diseases/conditions between those with and without confirmed COVID-19 (Supplementary Table S1 and S2 online).

### Prevalence of post COVID-19

In total, 112 (16.5%) of those with confirmed COVID-19 fulfilled the definition of post COVID-19 with no significant difference between females (17.8%) and males (14.5%). This prevalence was similar in the group of COVID-19 cases identified from the SmiNet register (16.3%, 58/356). When defining post COVID-19 as symptoms lasting for at least 3 months, the prevalence was 15.0% among those with confirmed COVID-19. Nineteen participants (2.8%) had been seeking healthcare for post COVID-19 and only 3 participants (0.4%) had received a diagnosis of post COVID-19 (Table 2). The overall prevalence of post COVID-19 in the total study population including both cases and non-cases of COVID-19 was 5.5% (112/2022).

**Table 2:** Prevalence of reported long-term symptoms, health care consumption and diagnosis of post COVID, by sex among participants with confirmed COVID-19^a^ (n = 681).

	Females (n = 405)	Males (n = 276)	Total (n = 681)	p-value^b^
Post COVID-19 symptoms ≥ 2 months, n (%)	72 (17.8)	40 (14.5)	112 (16.5)	0.26
Post COVID-19 symptoms ≥ 3 months, n (%)	66 (16.3)	36 (13.0)	102 (15.0)	0.24
Healthcare contact for post COVID-19 symptoms, n (%)	8 (2.0)	11 (4.0)	19 (2.8)	0.12
Post-COVID-19 diagnosis, n (%)	2 (0.5)	1 (0.4)	3 (0.4)	0.80
At least two post-COVID-19 symptoms (at least 1 symptom for ≥ 2 months)	48 (11.9)	27 (9.8)	75 (11.0)	0.40

^a^Confirmed COVID-19: self-reported SARS-CoV-2 positive PCR-test, antigen test or IgG test before vaccination and/or positive PCR-test in the database SmiNet (a national register covering all positive PCR-tests for SARS-CoV-2 conducted in Sweden).

^b^p-value obtained by Chi-2 test comparing the prevalence in females and males.

**Figure 1:**
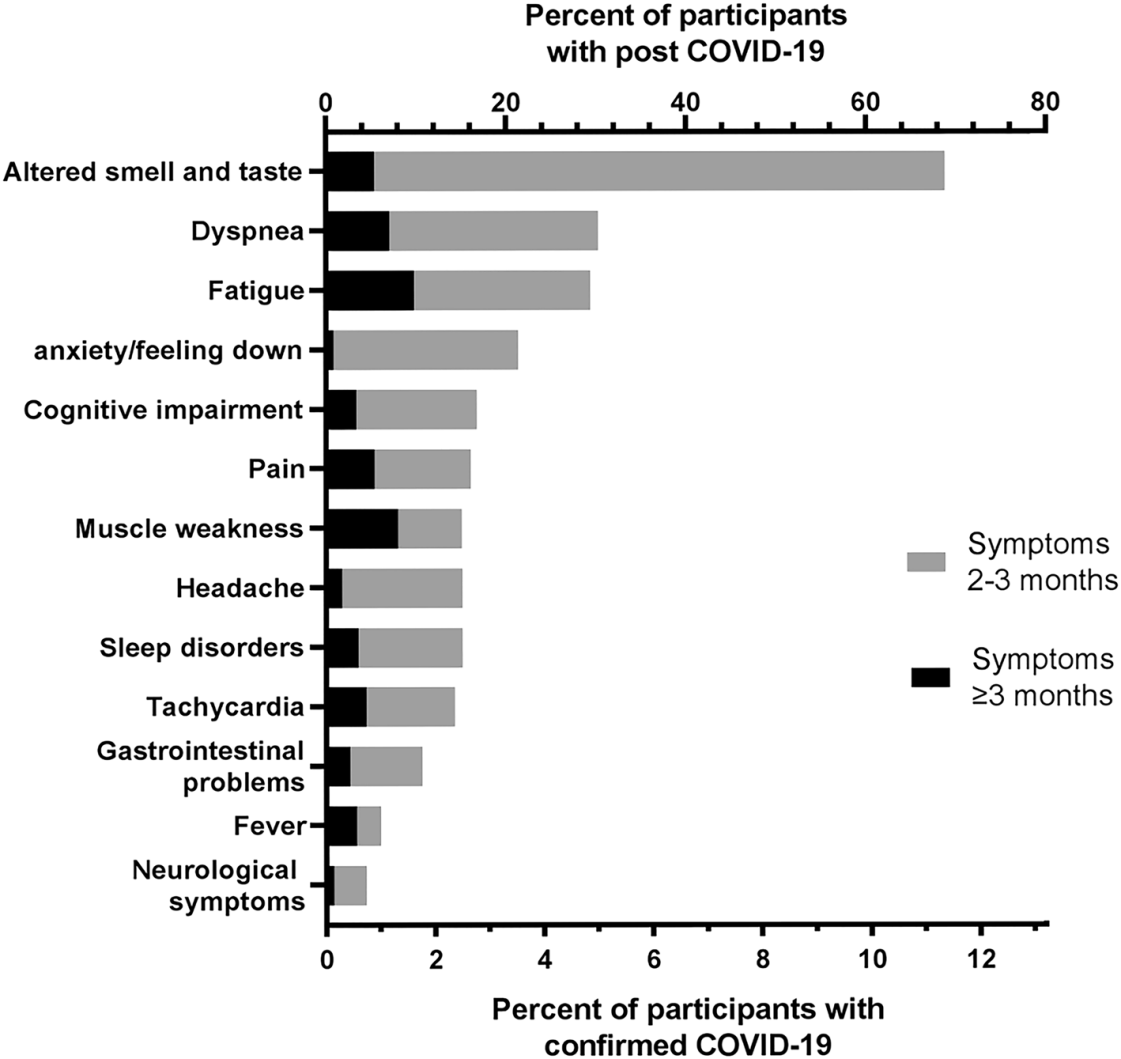
Prevalence of specific long-term symptoms among participants with confirmed COVID-19 and post COVID-19. Black part of the bars represents the participants with symptoms for at least two but less than three months, and the grey part represents the participants with symptoms for three months or more. The upper X-axis designates percent of participants with post COVID-19 (n = 112), the lower percent of participants with confirmed COVID-19 (n = 681).

Among those with post COVID-19 (n = 112), the most common symptoms were altered smell and taste (68.8%), dyspnea (33.7%) and fatigue (30.4%) (Fig. 1). There was no difference in type of symptoms between females and males (data not shown).

### Description of sociodemographic factors, lifestyle factors, and chronic diseases in relation to post COVID-19

**Figure 2:**
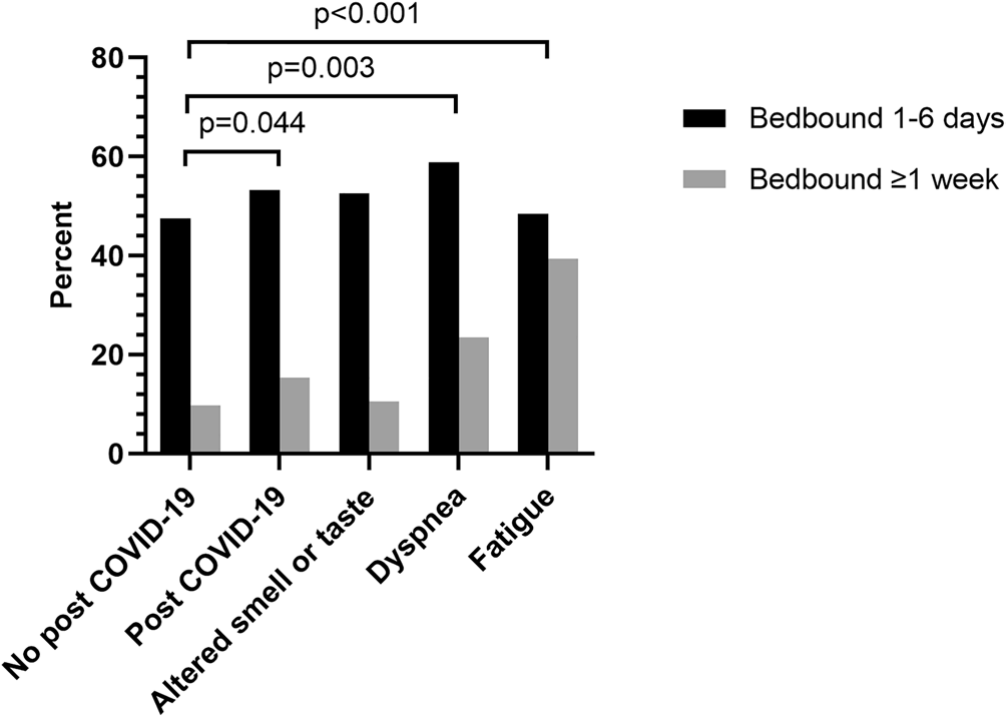
Percent of participants being bedbound during the COVID-19 disease in relation to post COVID-19. The figure shows the groups with and without post-COVID-19, and the groups with the three most prevalent specific symptoms of post COVID-19 (altered smell or taste, dyspnea, and fatigue). All groups are compared to the group with no post COVID-19, as three categories (not being bedbound, bedbound 1–6 days and bedbound ≥ 1 week). Significant differences are displayed in the figure.

There were no differences in sociodemographic factors (sex, education, occupation, or living area) among participants with confirmed COVID-19 with and without post COVID-19. Being bedbound during COVID-19, especially for one week or more, was more common among those with post COVID-19. This was more pronounced in the groups with symptoms of dyspnea and fatigue, Fig. 2 and Supplementary Table S3 online for exact numbers. There was a high vaccination coverage for COVID-19, at over 90% with at least one dose, not differing between the groups (data not shown).

**Figure 3:**
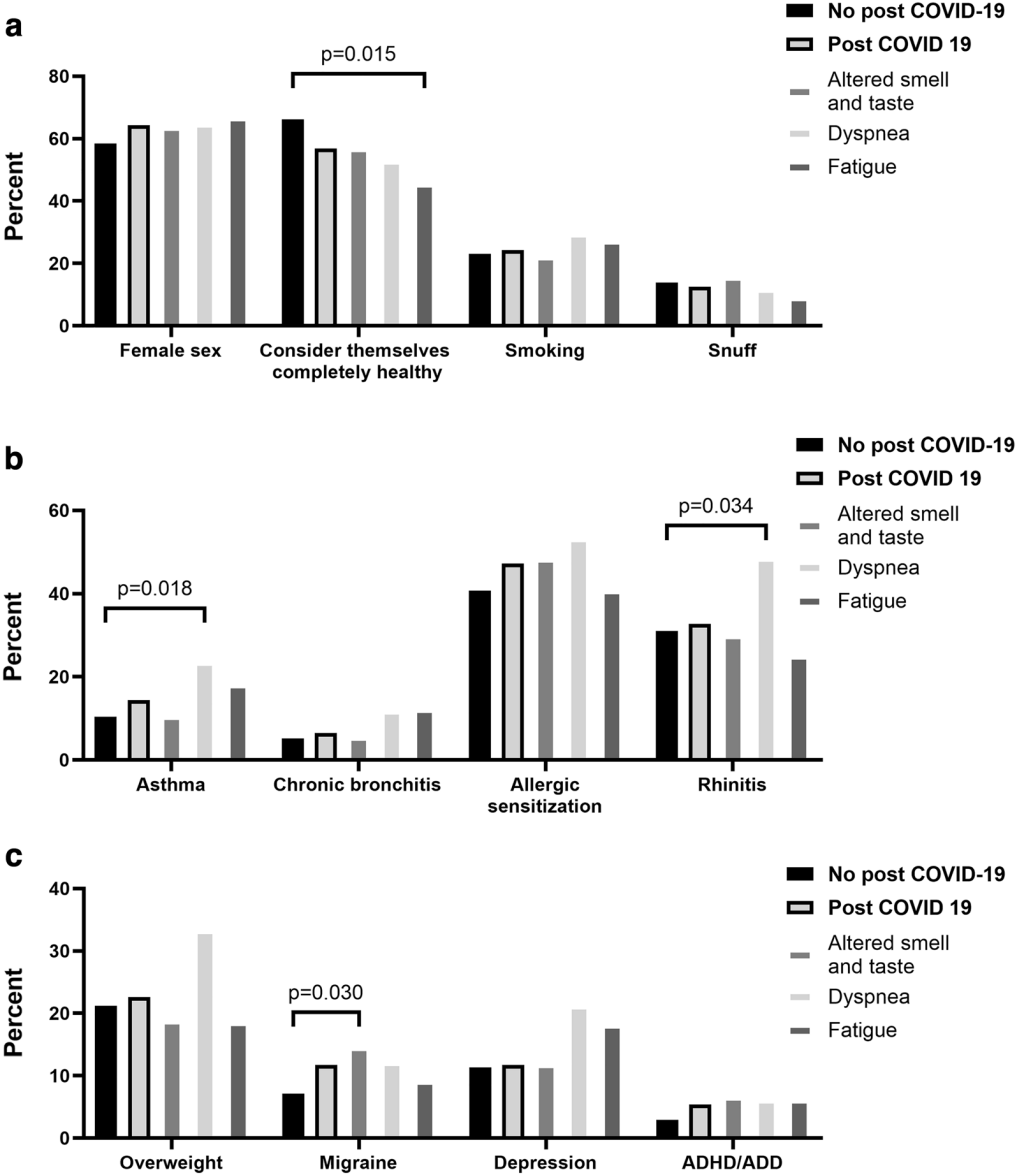
Prevalence of lifestyle factors, chronic diseases, and other characteristics before the pandemic in relation to post COVID-19. The figure shows the groups with and without post-COVID-19, and the groups with the three most prevalent specific symptoms of post COVID-19 (altered smell or taste, dyspnea, and fatigue). All groups are compared to the group with no post COVID-19. All exposures are assessed at the 24-year follow-up in 2016–2019.

Early life factors were not related to post COVID-19 or to the specific post COVID-19 symptoms altered taste or smell, dyspnea or fatigue (Supplementary Table S4 online). In addition, there was no significant difference in smoking or snuff use, assessed before the pandemic (Fig. 3A, Supplementary Table S4 online). However, the participants with post COVID-19 reported less often to have considered themselves completely healthy before the pandemic, which was more pronounced and significant in the group with post COVID-19 symptoms of fatigue (45.5% n = 15, compared to 66.2%, n = 370, among them with no post COVID-19, p = 0.015) (Fig. 3A).

Participants with post COVID-19 dyspnea also had a higher prevalence of asthma and rhinitis before the pandemic (Fig. 3B), and a higher prevalence of overweight, although not significant (Fig. 3C). Migraine was more common among participants with post COVID-19, significant in the group with altered taste and smell, while the prevalence of ADHD/ADD, chronic bronchitis and depression before the pandemic did not differ significantly (Fig. 3C). No difference in lung function (neither spirometry before or after bronchodilation nor lung clearance index (LCI) before the pandemic was observed in relation to post COVID-19 (Supplementary Table S5 and S6 online).

### Adjusted association analyses of potential risk factors for post COVID-19

In a mutually adjusted multivariable logistic regression analysis among participants with confirmed COVID-19 (n = 510), no significant associations were observed between sociodemographic factors, lifestyle factors or chronic diseases and post COVID-19 (Table 3). As observed in the descriptive analyses, being bedbound during COVID-19 for one week or more was related to an increased risk for post COVID-19, however the confidence interval was broad, and the association did not reach statistical significance (OR 1.69, 95% CI 0.81, 3.56).Similar results were observed when investigating post COVID-19 defined as symptoms lasting for at least 3 months after COVID-19 (Supplementary Table S7).

**Table 3:** Association between sociodemographic factors during the pandemic as well as chronic diseases before the pandemic and post COVID-19 among participants with confirmed COVID-19, analyzed by a mutually adjusted multivariable logistic regression analysis (n = 510).

	Post COVID-19	
	OR	95% CI
Sex		
Females	Referent	–
Males	1.00	0.62–1.64
Living area		
Outside of Stockholm County	Referent	–
Stockholm County	1.22	0.63–2.37
Education		
Elementary school, high school, or upper secondary school	Referent	–
University/College	0.89	0.54–1.45
Occupation		
Student	Referent	–
Worker	0.73	0.44–1.23
Other	1.01	0.39–2.63
Bedbound during COVID-19		
No	Referent	–
Yes, 1–6 days	1.30	0.78–2.17
Yes, ≥ 1 week	1.69	0.81–3.56
BMI status at 24 years		
Normal weight	Referent	–
Overweight	1.03	0.59–1.81
Smoking at 24 years		
No	Referent	–
Yes	0.95	0.54–1.66
Asthma at 24 years		
No	Referent	–
Yes	1.19	0.59–2.39
Consider themselves completely healthy		
No	Referent	–
Yes	0.67	0.42–1.08

## Discussion

In the present study based on data from a population-based cohort with young adults, we observed that post COVID-19 symptoms for two months or more were present among 16.5% of the participants with confirmed COVID-19. However, few of these had been seeking healthcare for post COVID-19 symptoms or received a diagnosis of post COVID-19. The most common symptom was an altered smell and taste, present in more than two thirds of those with post COVID-19, followed by dyspnea and fatigue. Compared to participants without post COVID-19, those with post COVID-19, especially post COVID-19 dyspnea and fatigue had more often been bedbound during the COVID-19 infection. We found no difference in the prevalence of post COVID-19 between the sexes and observed no major differences in lifestyle factors or chronic diseases linked to post COVID-19. However, having asthma and/or rhinitis before the pandemic were associated with post COVID-19 dyspnea, migraine with altered smell/taste, and lower self-rated health with fatigue.

The observed prevalence of post COVID-19 is lower than most previous studies^7,17,22,23^, although relatively similar to other population-based studies^15,26,27^. For example, data from the United States (US) Centers for Disease Control and Prevention estimated that 19% of those reported having COVID-19 in the past still had symptoms of long COVID at the time of the survey^26^. Another large cross-sectional study among US adults observed that 15% of the individuals with a positive COVID-19 test reported continued symptoms for more than 2 months after the acute illness^15^. However, a pooled analysis based on 54 studies and two medical record databases in the United States found a lower prevalence; 6.2% of the individuals with symptomatic SARS-CoV-2 infections developed at least one of three self-reported long COVID symptom clusters three months after symptomatic SARS-CoV-2 infection^6^. In our BAMSE cohort, we have previously investigated self-reported long-term symptoms of suspected COVID-19 during the first half year of the of the pandemic (February to August 2020)^16^. This study showed that 11% of the participants with suspected COVID-19 symptoms had long-term symptoms for at least 4 weeks. However, in the beginning of the pandemic, testing for COVID-19 was not yet available for the general public in Sweden, and therefore, we were not able to investigate confirmed disease at this point.

Previous studies have identified older age as a risk factor for post COVID-19^4,13^, however^28^, few studies have focused specifically on young adults. In children, the prevalence of post COVID has been shown to be lower compared to adults, however a substantial number of children have been reported to be affected^6,29^. As in adults, a wide range of long-term symptoms have been reported in children, although with a higher prevalence of mental health problems^28,29^.

In contrast to most previous studies in adults^9^, we did not observe a significant over representation of females with post COVID-19, although there was a slightly higher prevalence among the females. The sex distribution of post COVID-19 has been found to differ depending on age with women more commonly affected in adulthood and up to approximately 60 years of age^30^, whereas men are over-represented in the older age groups^4^.

After a severe COVID-19 disease, breathing difficulties has been shown to be the most common post COVID-19 symptom^21^, while a disease treated in primary care was more often associated with tiredness/fatigue^22–24^. The most common post COVID-19 symptom in our cohort was altered taste and smell, present in 69% of the participants with post COVID-19 (11.3% of the participants with confirmed COVID-19). This is a higher prevalence than in most outpatient populations investigated^4,22,23,31^, although a prevalent symptom in the acute phase of the disease^32,33^. In some studies, olfactory dysfunction has been linked to mild COVID-19^34^, a finding in line with our results where dyspnea and fatigue, but not altered taste/smell, was linked to disease severity (being bedbound). In a recent large Norwegian cohort study including participants aged 35–65 years, altered smell and taste was found to be the most specific symptom for post COVID-19. The other symptoms could be grouped in two clusters, one with symptoms such as fatigue and poor memory, and one related to respiratory symptoms like dyspnea and cough, suggesting different aetiologies^35^. This study also found more severe disease to be associated to a higher risk for post COVID-19 symptoms in the two clusters mentioned^35^. Together with previous studies, our results support an association between disease severity and spectrum of post COVID-19 symptoms.

In the present study, several potential risk factors for post COVID-19 were investigated. We did not find early life factors including infections or respiratory symptoms to be associated with post COVID-19. Among the conditions reported at 24 years of age, there were no significant associations between lifestyle (tobacco use) or chronic conditions/diseases and post COVID-19, although both rhinitis and asthma were associated with post COVID-19 dyspnea. However, no association was found between lung function at 24 years of age and post COVID-19, neither spirometric nor measured with MBWO. COVID-19 in turn has not been shown to affect spirometric lung function in young adults with mild disease^36^. This, however, does not rule out other conditions in the lung, such as a decreased diffusion capacity, found to be a sequela often proportionate to disease severity COVID-19^37,38^.

Overweight was not related to post COVID-19 but was more common among those with post COVID-19 dyspnea (although the difference was not significant). These results are in line with the previously established link between overweight and an altered ventilation pattern as well as chronic inflammation and asthma^39^. In addition, we have previously reported an association between asthma and higher levels of concern of the own health and stress associated to COVID-19^40^, in all, suggesting the dyspnea in the post COVID-19 context to be a multifactorial condition.

Previous studies have observed that co-morbidities increase the risk for post COVID-19^38^. In the present study, we did not find doctors diagnosed depression, ADHD or ADD, to have any significant association to post COVID-19, although doctors diagnosed migraine was more common among the participants with altered taste or smell. However, the lower level of participants who reported to be “completely healthy” at 24 years of age may suggest that those with post COVID-19 symptoms have an increased susceptibility or other pre-existing diseases that were not assessed in this study.

A limitation with our study is that some of the confirmed COVID-19 cases were self-reported (based on positive PCR, antigen, or antibody test taken before vaccination). The antigen tests have been shown to be less accurate compared to PCR-tests, although with a relatively high positive predictive value (true positive)^41^. Also, not all participants may have been tested for COVID-19, especially those with mild disease, which may lead to an incorrect estimate of the true proportion of post COVID-19. A general limitation in investigation of post COVID-19 is the unspecific character of the symptoms, which may be attributed to conditions other than COVID-19^42^. As in all questionnaire-based studies, there is also a risk of recall bias when reporting previous symptoms. In addition, there was no question on whether the participants still had symptoms at the time of answering the questionnaire and therefore no possibility to investigate how many that eventually recovered from their post COVID. It is also shown that post COVID symptoms differs by viral variant^15,43^, and the results may therefore not be representative of current viral variants. Unfortunately, there was no available information about which viral variant the participants were infected with, and this could therefore not be investigated further. With regards to the time point of data collection, this study likely mostly included wild-type SARS-CoV-2 (for cases during 2020), the alpha-variant (dominated in Sweden in the first half of 2021) and the delta variant (dominated in Sweden in the second half of 2021)^44^.

The main strength of the study is the population-based design with a well-characterized study-population. Information on lifestyle factors and chronic diseases had been collected recently before the pandemic, as well as in early life. Another strength is the high response rate limiting the risk of selection bias, although this cannot be ruled out since participants with post COVID-19 symptoms may be more prone to participate in a COVID-19 directed follow-up. At the same time, it is also possible that the most severe cases to a lower extent answered the questionnaire, which may have led to an underestimation of severe post COVID.

In conclusion, post COVID-19 symptoms are common, also among young adults in the general population. We identified no major risk factors including lifestyle and chronic diseases present before the pandemic for being affected, suggesting other factors and mechanisms to be involved (including genetics and immune response)^45^. Although not life threatening, post COVID-19 could have a considerable impact on public health due to the high prevalence and long-term symptoms.

## Methods

**Figure 4:**
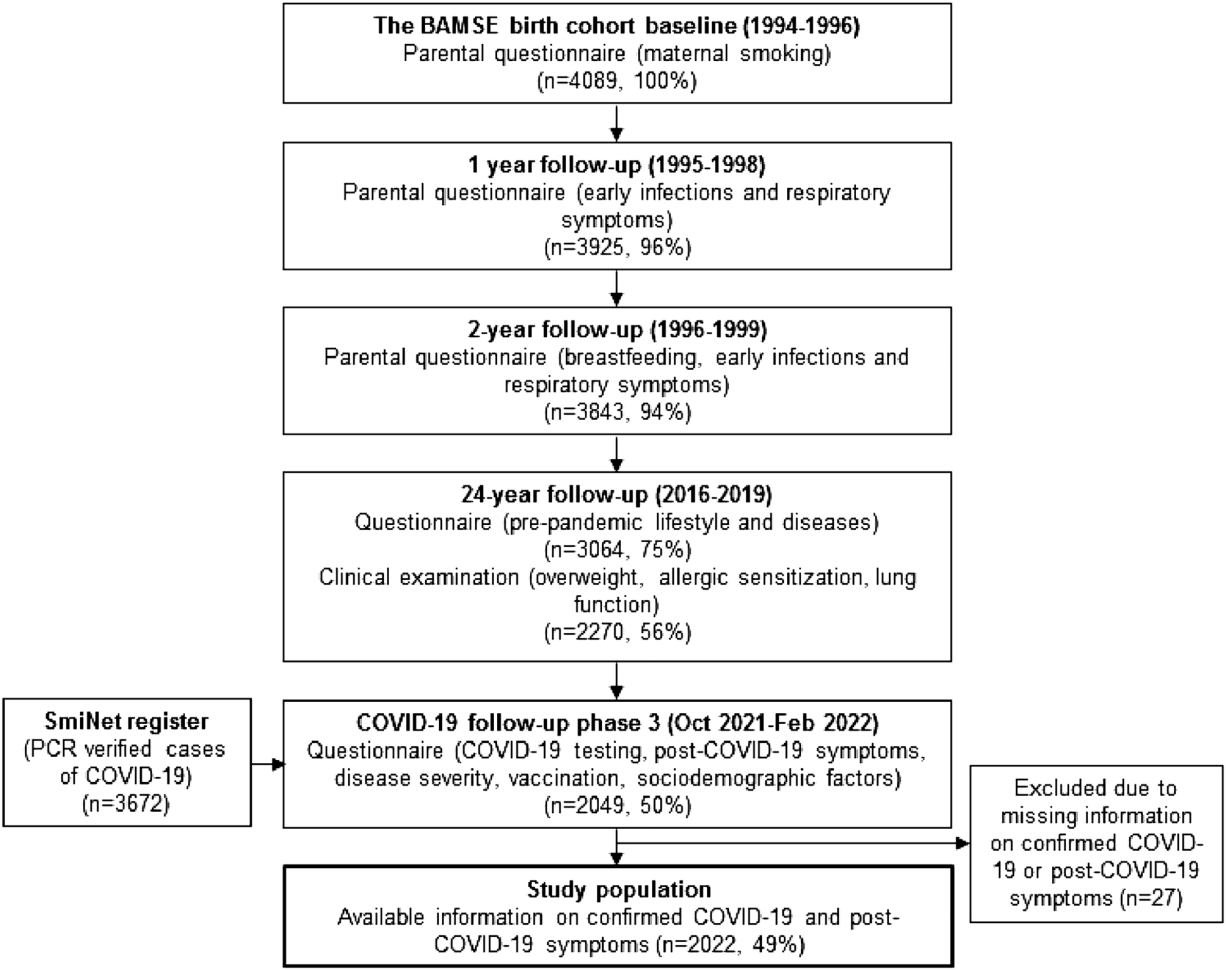
Flow chart of the included study population.

The included population came from the population-based birth cohort BAMSE (Swedish: *Barn, Allergi, Miljö, Stockholm, Epidemiologi*). The cohort was initiated 1994–1996 including originally 4089 children born in pre-selected areas of Stockholm County and have been followed by repeated questionnaires and examinations to approximately 26 years of age so far. The participants parents responded to questionnaires regarding early exposures at inclusion at 2–3 months and 1 and 2 years of age^46^. At 22–24 years of age (2016–2019), the participants were followed-up with questionnaires and a physical examination. After the onset of the COVID-19 pandemic, further investigations have been done in three phases including repeated web-questionnaires on COVID-19 symptoms, blood sampling and a clinical investigation^47^. For this investigation questionnaire data from the third phase conducted between October 2021 and February 2022 was used. Invitation to the third phase was sent to all participants who had participated in the questionnaire at the 22–24 years follow up and had provided their e-mail address (n = 2981). In total, 2049 (68.7%) participated by answering a web-based questionnaire including questions on post COVID-19 symptoms, lifestyle, and health during the whole pandemic (from February 2020 up to the date of the questionnaire) (Fig. 4). Due to missing data on confirmed COVID-19 disease and/or post COVID-19 symptoms, the final study population consisted of 2022 participants (Fig. 4). The BAMSE-study is approved by the Ethical Review Board at Karolinska Institute Dnr: 93:189; Dnr: 2016/1380-31/2; Dnr: 2016/2475-32; Dnr: 2020-02922. Written informed consent was obtained from all participants and/or their legal guardians. All research was performed in accordance with the Declaration of Helsinki.

### Definition of COVID-19

Confirmed COVID-19 was defined as any of: self-reported SARS-CoV-2 positive PCR-test, antigen test or IgG test (IgG taken before vaccination) and/or positive PCR-test in the database SmiNet (a national register for notifiable transmittable diseases in Sweden, covering all positive PCR-tests)^25,48^. up to the date of the questionnaire. Being bedbound during the disease was used as a proxy for disease severity.

### Definition of post COVID-19 and post COVID-19 symptoms

Participants with confirmed or suspected COVID-19 were asked about long-term symptoms after the COVID-19 infection “Have you had long term symptoms after the COVID-19 infection (post COVID/long COVID)?” Participants who answered “Yes” to this question were asked about which long-term symptoms they have had and whether the duration of each symptom was two months or more or three months or more. The included long-term symptoms were: dyspnea (breathing difficulties or shortness of breath); fatigue (extreme physical and/or mental tiredness); fever (or feeling feverish); altered smell or taste; headache; tachycardia (high resting heart rate or palpitations); cognitive impairment (e.g., memory and concentration difficulties); gastrointestinal problems; muscle weakness; neurological symptoms (e.g. numbness); psychiatric symptoms (e.g. depression, anxiety or feeling down); pain (e.g., chest pain or muscle and joint pain) and sleep disorders.

Post COVID-19 was defined as at least one symptom lasting for at least 2 months after COVID-19 in combination with confirmed COVID-19.

### Description of risk factors and background characteristics

Information on occupation, disease severity during COVID-19 and vaccination status was collected from the phase three COVID-19 follow up. In Sweden, vaccination against COVID-19 was offered to young adults around the summer of 2021, with some variations depending on county. Pre-existing risk factors were collected from the 24-year follow up and early exposures from the baseline questionnaire, 1- and 2-years questionnaires.

Early factors investigated were maternal smoking during pregnancy and/or infancy, pneumonia, wheeze and bronchitis up to 2 years of age. At the follow-up at 24 years of age the following exposures were evaluated in association to post COVID-19: smoking and snuff use, overweight, asthma, allergic sensitization, rhinitis, and self-reported doctors diagnosed (ever) migraine, ADHD or ADD, depression and self-reported health.

At the 24-year follow-up lung function testing was done with spirometry before and after bronchodilation, and multiple breath nitrogen wash out test (MBWO), according to the ATS/ERS guidelines^49,50^. The details and definitions of the included variables are described in the Online Supplement.

### Statistical analyses and study design

The study population was characterized regarding sociodemographic factors and background factors, and differences between the groups (e.g., with and without confirmed COVID-19) were tested by Chi-squared or Fisher’s exact test. The prevalence of post COVID-19 was calculated among those with confirmed COVID-19 and stratified by sex. The prevalence of specific post COVID-19 symptoms was presented among those with post COVID-19 and among all participants with confirmed COVID-19.

Potential risk factors for post COVID-19 were investigated by comparing sociodemographic factors, early exposures, lifestyle factors and chronic diseases/conditions at the 24-year follow-up between participants with and without post COVID-19 (among those with confirmed COVID-19). The three most common post COVID-19 symptoms were thereafter analysed separately in a similar way. Lung function was compared between the post COVID-19 and no post COVID-19 groups as a continuous variable with two-tailed t-tests. Associations between sociodemographic- and lifestyle factors as well as chronic diseases before the pandemic and post COVID-19 were further analyzed by a mutually adjusted multivariable logistic regression analysis in a complete case analysis. The included variables were selected based on previously identified risk factors for post COVID-19 as well as from the descriptive analyses. As a sensitivity analysis, post COVID-19 was also defined as symptoms lasting for at least 3 months after COVID-19 in combination with confirmed COVID-19.

The analyses were done in STATA, Statistical Software release 16.0 (College Station, TX, USA), and diagrams/Figures in GraphPad Prism 9.3.0 (San Diego, CA, USA). Inkscape 0.92 (http://www.inkscape.org) and Microsoft Power Point (version 2203), were used for creating Figures. A 2-sided p-value < 0.05 was considered statistically significant.

## Supplementary Information

Supplementary Information.

## Data Availability

The datasets generated and/or analyzed during the current study are not publicly available due to the dataset containing sensitive personal data but are available from the corresponding author on reasonable request and with permission from Karolinska Institutet.

## Code availability

The computer code used to generate the results can be made available upon request.
