# Supplementary material for: Post COVID-19 symptoms are common, also among young adults in the general population
Source: Sci Rep. 2023 Jul 12;13:11300. doi: 10.1038/s41598-023-38315-2 (PMC10338459; doi:10.1038/s41598-023-38315-2)
Supplement: Supplementary file 1 — Supplementary Information. [file 41598_2023_38315_MOESM1_ESM.pdf]

Online supplement

Post COVID-19 symptoms are common, also among young adults in the general population

Ida Mogensen, Sandra Ekström*, Jenny Hallberg, Antonios Georgelis, Erik Melén, Anna Bergström, Inger Kull

The first 2 authors contributed equally to this article, and both should be considered first author. The last 2 authors contributed equally to this article, and both should be considered senior author.

*corresponding author

BAMSE COVID-19 Study Group:

Catarina Almqvist, Niklas Andersson, Natalia Ballardini, Anna Bergström, Sophia Björkander, Petter Brodin, Anna Castel, Sandra Ekström, Antonios Georgelis, Jenny Hallberg, Lennart Hammarström, Qiang Pan-Hammarström, Christer Janson, Maura Kere, Inger Kull, André Lauber, Alexandra Lövquist, Erik Melén, Jenny Mjösberg, Ida Mogensen, Lena Palmberg, Göran Pershagen, Chris Tibbitt, Niclas Roxhed, Jochen M. Schwenk

Definition of co-variables and chronic diseases

Baseline questionnaire

Maternal smoking: the mother smoked at least one cigarette per day at any point of time during pregnancy and/or in infancy

1–2-year follow-up questionnaire

Bronchitis first 2 years: parental-reported doctor's diagnosis of bronchitis reported in the 1-year and/or 2-year questionnaire

Pneumonia first 2 years: parental-reported doctor's diagnosis of pneumonia reported in the 1-year and/or 2-year questionnaire

Wheeze first 2 years: at least one episode of wheeze (after 3 months of age) reported in the 1-year and/or 2-year questionnaire

24-year follow-up questionnaire

Smoking: self-reported based on the question "do you smoke", with the answer alternatives 'No', 'No, but I have smoked before' (referred to as ex-smoker), 'Yes, sometimes', 'Yes, every day'. Smoking was also categorized as yes/no in some analyses due to low numbers.

Snuff use: self-reported based on the question "do you use snuff", with the answer alternatives 'No', 'No, but I have used snuff before' (referred to as ex-user), 'Yes, sometimes', 'Yes, every day'. Snuff use was also categorized as yes/no in some analyses due to low numbers.

Asthma: doctor's diagnosis of asthma (ever) together with symptoms of breathing difficulties or asthma medication occasionally or regularly in the last 12 months.

Chronic bronchitis: cough and mucus production in the morning during winter.

Rhinitis: symptoms from eyes or nose after exposure to furred animals or pollen (without having a flu) in the last 12 months

Migraine, Depression and ADHD or ADD: self-reported doctor's diagnosis (ever) of "Migraine", "Depression", "ADHD or ADD", respectively.

Consider themselves completely health: answering "Completely healthy" to the question "How healthy would you say that you are?"

24-year follow-up examination

Overweight: defined from measured weight and height as a body mass index ≥ 25 kg/m^2.

Sensitization to airborne allergens were analyzed from the blood samples using the Phadiatop mix (cat, dog, horse, timothy, birch, mug wort, house dust mite and mold). IgE values ≥ 0.35kU/L were considered positive.

Lung function: lung function testing was performed with spirometry (Vyaire Vyntus system, Vyair Medical, IL, USA, according to the ATS/ERS guidelines [1]; bronchodilation with four puffs of Salbutamol in spacer with new measurements 15 minutes later). Measured values were expressed as precent of predicted (pp) using the Global Lung Initiative reference

material[2]. Multiple breath nitrogen wash out test was performed at least twice with an Exhalyzer D (Ecomedics Technologies™, San Diego, CA) according to ERS guidelines [3]. As software, Spiroware 3.2.0 was used, and migrated to Spiroware 3.3.1 to correct for previously reported calculation errors [4-6].

COVID-19 follow-up questionnaire

Education: self-reported highest completed education at the time of the phase 3 questionnaire. Categorized as elementary school, high school/upper secondary school, university/College <3 years/folk high school and university ≥ 3 years.

Occupation: self-reported occupation at the time of the phase 3 questionnaire and categorized as studying, working, other (including the answer alternatives “parental leave or unpaid leave”, “unemployed”, “furloughed due to the current pandemic” or “other”)

Living area: based on the registered postal address at the time of questionnaire 3, categorized as Stockholm County or outside of Stockholm County

Bedbound during COVID-19: bedbound during the COVID-19 infection with the answer alternatives “no”, “yes, 1-6 days”, “yes, ≥ 1 week”

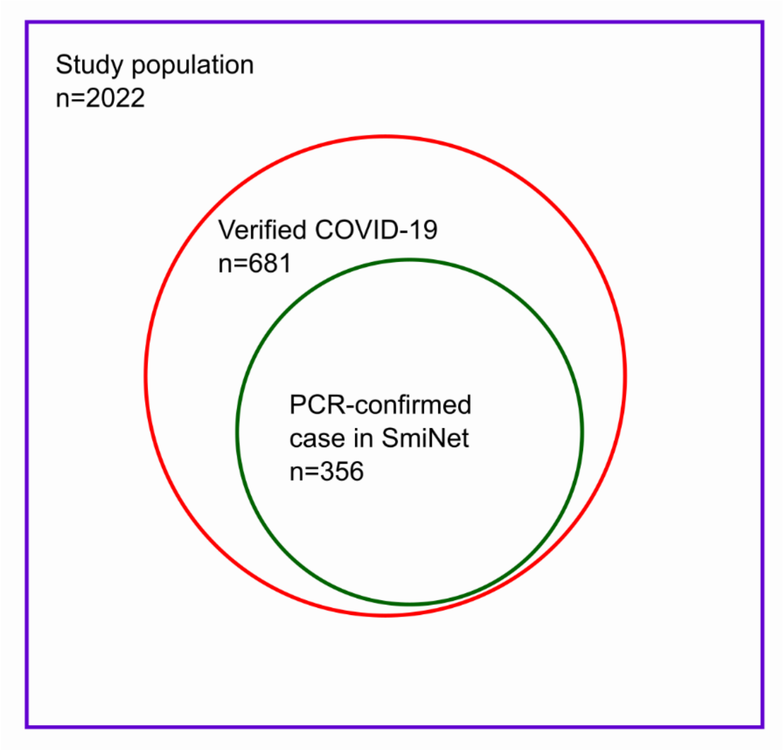

Supplementary Figure 1. Proportional Venn-diagram showing PCR-confirmed cases in the SmiNet register (a national register covering all positive PCR-tests for SARS-CoV-2 conducted in Sweden) and total number of confirmed cases of COVID-19 (PCR-verified in SmiNet and/or reported positive PCR and/or reported positive antigen test and/or reported IgG if unvaccinated during the clinical examination). Figure created in Stata and Inkscape.

Supplementary Table S1. Description of sociodemographic factors, disease severity and vaccination against COVID-19 among participants with and without confirmed COVID-19[1] (n=2022)

	**No confirmed COVID-19 (n=1341)**	**Confirmed COVID-19 (n=681)**	
	**n (%)**	**n (%)**	**P-value[2]**
**Sex (n=2022)**			0.76
- Female	788 (58.8)	405 (59.5)	
- Male	553 (41.2)	276 (40.5)	
**COVID-19 follow-up variables**	**n (%)**	**n (%)**	**P-value[2]**
**Education (n=1948)**			0.68
- Elementary school	26 (2.0)	11 (1.7)	
- High school/upper secondary school	373 (28.7)	184 (28.4)	
- University/College <3 years or folk high school	194 (14.9)	110 (17.0)	
- University ≥ 3 years	706 (54.4)	344 (53.0)	
**Occupation (n=1953)**			0.79
- Studying	364 (27.9)	183 (28.0)	
- Working	844 (64.8)	426 (65.5)	
- Other	95 (7.3)	42 (6.5)	
**Living area (n=2022)**			**<0.001**
- Stockholm County	**986 (73.5)**	**565 (83.0)**	
- Outside of Stockholm County	**355 (26.5)**	**116 (17.0)**	

[1] Confirmed COVID-19 self-reported SARS-CoV-2 positive PCR-test, antigen test or IgG test before vaccination and/or positive PCR-test in the database SmiNet (a national register covering all positive PCR-tests for SARS-CoV-2 conducted in Sweden).
[2] P-value obtained by chi-2 test

Supplementary Table S2. Description lifestyle and chronic diseases before the pandemic among participants with and without confirmed COVID-19[1] (n=2022)

	**No confirmed COVID-19 (n=1341)**	**Confirmed COVID-19 (n=681)**	
Early factors (1-2 year questionnaire)	**n (%)**	**n (%)**	**P-value[2]**
Maternal smoking in pregnancy and/or infancy (n=2021)	151 (11.3)	94 (13.8)	0.10
Pneumonia first 2 years (n=1948)	100 (7.8)	49 (7.5)	0.81
Wheeze first 2 years (n=1948)	315 (24.4)	180 (27.3)	0.17
Bronchitis first 2 years (n=1943)	180 (14.0)	104 (15.8)	0.28
24- year follow-up variables	**n (%)**	**n (%)**	**P-value[2]**
Smoking (n=2018)			<0.001
- No	960 (71.8)	430 (63.1)	
- Ex-smoker	151 (11.3)	97 (14.2)	
- Yes, sometimes	134 (10.0)	113 (16.6)	
- Yes, everyday	92 (6.9)	41 (6.0)	
Snuff use (n=2020)			0.20
- No	1117 (83.4)	560 (82.2)	
- Ex-user	35 (2.6)	28 (4.1)	
- Yes, sometimes	54 (4.0)	33 (4.9)	
- Yes, everyday	133 (9.9)	60 (8.8)	
Overweight (n=1675)	239 (21.7)	125 (21.7)	1.00
Asthma (n=2017)	154 (11.5)	75 (11.1)	0.76
Chronic bronchitis (n=1972)	71 (5.4)	36 (5.5)	0.98
Rhinitis (n=2001)	408 (30.8)	210 (31.1)	0.88
Sensitization to airborne allergens (n=1656)	472 (43.5)	240 (42.1)	0.60
Migraine doctors diagnosed (n=1970)	**74 (5.7)**	**53 (8.0)**	**0.049**
Depression doctors diagnosed (n=1971)	**202 (15.5)**	**75 (11.3)**	**0.01**
ADHD or ADD doctors diagnosed (n=1973)	**74 (5.7)**	**22 (3.3)**	**0.02**
Consider themselves completely healthy (n=1981)	823 (62.7)	432 (64.7)	0.39

[1] Confirmed COVID-19: self-reported SARS-CoV-2 positive PCR-test, antigen test or IgG test before vaccination and/or positive PCR-test in the database SmiNet (a national register covering all positive PCR-tests for SARS-CoV-2 conducted in Sweden).
[2] P-value obtained by chi-2 test

Supplementary Table S3. Description of disease severity (being bedbound during the COVID-19 disease) among participants with confirmed COVID-19 with and without post COVID-19, as well as for the groups with the three most common specific symptoms of post COVID-19 (altered smell and taste, dyspnea, and fatigue).

	**No post COVID-19 (n=518)**	**Post COVID-19 (n=112)**	**Altered smell/taste (n=76)**	**Dyspnea (n=34)**	**Fatigue (n=33)**
	n (%)	n (%)	n (%)	n (%)	n (%)
**Bedbound during disease (n=)**					
- No	222 (42.9)	35 (31.5)	28 (36.8)	6 (17.7)	4 (12.1)
- Yes, 1-6 days	246 (47.5)	59 (53.2)	40 (52.6)	20 (58.8)	16 (48.5)
- Yes, ≥ 1 week	50 (9.7)	17 (15.3)	8 (10.5)	8 (23.5)	13 (39.4)
P-value[1]		0.044	0.61	0.003	<0.001

[1] All groups as compared to the groups with no post COVID-19 symptoms using chi-2 test.

Supplementary Table S4. Description lifestyle and chronic diseases before the pandemic (at the 24-year follow-up among participants with confirmed COVID-19 with or without post COVID-19

	Confirmed COVID-19[1] without post COVID (n=569)	Confirmed COVID-19[1] with post COVID-19 (n=112)	
**Early factors (1–2-year follow-up)**	**n (%)**	**n (%)**	**P-value[2]**
Maternal smoking in pregnancy and/or infancy (n=681)	76 (13.4)	18 (16.1)	0.45
Pneumonia first 2 years (n=658)	37 (6.7)	12 (11.2)	0.11
Wheeze first 2 years (n=659)	154 (27.9)	26 (24.5)	0.48
Bronchitis first 2 years (n=657)	90 (16.3)	14 (13.2)	0.42
**24- year-follow-up variables**	**n (%)**	**n (%)**	**P-value[2]**
Smoking (n=681)	128 (22.5)	26 (23.3)	0.87
Snuff use (n=681)	79 (13.9)	14 (12.5)	0.70
Overweight (n=575)	104 (21.6)	21 (22.6)	0.83
Asthma (n=678)	59 (10.4)	16 (14.4)	0.22
Chronic bronchitis (n=661)	29 (5.2)	7 (6.5)	0.60
Rhinitis (n=675)	174 (30.8)	36 (32.7)	0.69
Sensitization to airborne allergens (n=570)	196 (41.1)	44 (47.3)	0.27
Migraine doctors diagnosed (n=665)	40 (7.2)	13 (11.7)	0.11
Depression doctors diagnosed (n=666)	62 (11.2)	13 (11.7)	0.87
ADHD or ADD doctors diagnosed (n=666)	16 (2.9)	6 (5.4)	0.18
Consider themselves completely healthy (n=668)	370 (66.2)	62 (56.9)	0.06

[1] Confirmed COVID-19: confirmed case in SmiNet (PCR-verified) and/or reported positive PCR and/or reported positive antigen test and/or reported IgG if unvaccinated during the clinical examination (all from the questionnaire in phase 3)

[2] P-value obtained by chi-2 test or Fischer's exact test depending on size of the groups

Supplementary Table S5. Description of lung function before the pandemic (at the 24-year follow-up) among participants with confirmed COVID-19 with or without post COVID-19

	**No post COVID-19 Pre BD: n=440 Post BD: n=420**	**Post COVID-19 Pre BD: n=84 Post BD: n=79**	**P-value[1]**
	**mean (SD)**	**mean (SD)**	
**FEV1pp pre BD**	97.0 (9.5)	97.1 (9.9)	0.93
**FEV1pp post BD**	100.4 (9.3)	101.0 (9.8)	0.59
**FVCpp pre BD**	100.1 (9.8)	100.3 (9.8)	0.86
**FVCpp post BD**	99.6 (9.9)	100.5 (9.9)	0.44
**FEV1/FVC pre BD**	0.83 (0.058)	0.83 (0.064)	0.94
**FEV1/FVC post BD**	0.86 (0.049)	0.86 (0.056)	0.72
	n=246	N=51	
**LCI**	6.09 (0.41)	6.03 (0.37)	0.31

[1] P-value obtained by the t-test

BD: Bronchodilation; pp: percent of predicted; FEV1: forced exhaled volume the first second; FVC: forced vital capacity; LCI: lung clearance index

Supplementary Table S6. Description of lung function before the pandemic (at the 24-year follow-up) in participants without post COVID-19 compared with participants with post COVID-19 symptom of dyspnea

	No post COVID-19<br><br>Pre BD: n=440<br>Post BD: n=420	No post COVID-19 dyspnea<br>Pre BD: n=28<br>Post BD: n=25	P-value[1]
	**mean (SD)**	**mean (SD)**	
**FEV1pp pre BD**	97.0 (9.5)	97.3 (9.0)	0.86
**FEV1pp post BD**	100.4 (9.3)	100.9 (10.2)	0.79
**FVCpp pre BD**	100.1 (9.8)	101.5 (10.3)	0.45
**FVCpp post BD**	99.6 (9.9)	102.3 (10.6)	0.19
**FEV1/FVC pre BD**	0.83 (0.058)	0.82 (0.068)	0.51
**FEV1/FVC post BD**	0.86 (0.049)	0.85 (0.059)	0.088
	n=246	n=23	
**LCI**	6.09 (0.41)	6.15 (0.40)	0.50

Pre: n=440+28 Post: n=420+25

[1] P-value obtained by the t-test

BD: Bronchodilation; pp: percent of predicted; FEV1: forced exhaled volume the first second; FVC: forced vital capacity; LCI: lung clearance index

Supplementary Table S7. Association between sociodemographic factors during the pandemic as well as chronic diseases before the pandemic and post COVID-19 defined as symptoms lasting for at least 3 months after COVID-19 in combination with confirmed COVID-19, analyzed by a mutually adjusted multivariable logistic regression analysis (n=510)

	**Post COVID-19**	
	**OR**	**95% CI**
**Sex**		
Females	Referent	-
Males	0.93	0.56-1.56
**Living area**		
Outside of Stockholm County	Referent	-
Stockholm County	1.04	0.53-2.05
**Education**		
Elementary school, high school, or upper secondary school	Referent	-
University/College	0.78	0.47-1.29
**Occupation**		
Student	Referent	-
Worker	0.67	0.39-1.14
Other	0.87	0.32-2.38
**Bedbound during COVID-19**		
No	Referent	-
Yes, 1-6 days	1.27	0.74-2.16
Yes, ≥ 1 week	1.57	0.72-3.45
**BMI status at 24 years**		
Normal weight	Referent	-
Overweight	0.80	0.43-1.49
**Smoking at 24 years**		
No	Referent	-
Yes	0.82	0.45-1.50
**Asthma at 24 years**		
No	Referent	-
Yes	1.09	0.52-2.31
**Consider themselves completely healthy**		
No	Referent	-
Yes	0.62	0.38-1.01

References:

1 Graham, B. L. *et al.* Standardization of Spirometry 2019 Update. An Official American Thoracic Society and European Respiratory Society Technical Statement. *Am J Respir Crit Care Med* **200**, e70-e88, doi:10.1164/rccm.201908-1590ST (2019).

2 Quanjer, P. H. *et al.* Multi-ethnic reference values for spirometry for the 3-95-yr age range: the global lung function 2012 equations. *The European respiratory journal* **40**, 1324-1343, doi:10.1183/09031936.00080312 (2012).

3 Robinson, P. D. *et al.* Consensus statement for inert gas washout measurement using multiple- and single- breath tests. *The European respiratory journal* **41**, 507-522, doi:10.1183/09031936.00069712 (2013).

4 Wyler, F., Oestreich, M. A., Frauchiger, B. S., Ramsey, K. A. & Latzin, P. Correction of sensor crosstalk error in Exhalyzer D multiple-breath washout device significantly impacts outcomes in children with cystic fibrosis. *Journal of applied physiology (Bethesda, Md. : 1985)* **131**, 1148-1156, doi:10.1152/japplphysiol.00338.2021 (2021).

5 R, M. S., Gustafsson, P. M., Lindblad, A., Robinson, P. D. & K, G. N. Improved agreement between N(2) and SF(6) multiple-breath washout in healthy infants and toddlers with improved EXHALYZER D sensor performance. *Journal of applied physiology (Bethesda, Md. : 1985)* **131**, 107-118, doi:10.1152/japplphysiol.00129.2021 (2021).

6 Frauchiger, B. S. *et al.* Do clinimetric properties of LCI change after correction of signal processing? *Pediatric pulmonology*, doi:10.1002/ppul.25865 (2022).
